# FTIR spectroscopy as a unified method for simultaneous analysis of intra- and extracellular metabolites in high-throughput screening of microbial bioprocesses

**DOI:** 10.1186/s12934-017-0817-3

**Published:** 2017-11-13

**Authors:** Gergely Kosa, Volha Shapaval, Achim Kohler, Boris Zimmermann

**Affiliations:** 1 Faculty of Science and Technology, Norwegian University of Life Sciences, Postbox 5003, 1432 Ås, Norway; 2 Nofima AS, Osloveien 1, 1430 Ås, Norway

**Keywords:** Microcultivation, Oleaginous fungi, Citric acid, High-throughput screening, Fourier transform infrared spectroscopy, Partial least squares regression, Bioprocess monitoring, *Mucor*, *Umbelopsis*, *Penicillium*

## Abstract

**Background:**

Analyses of substrate and metabolites are often bottleneck activities in high-throughput screening of microbial bioprocesses. We have assessed Fourier transform infrared spectroscopy (FTIR), in combination with high throughput micro-bioreactors and multivariate statistical analyses, for analysis of metabolites in high-throughput screening of microbial bioprocesses. In our previous study, we have demonstrated that high-throughput (HTS) FTIR can be used for estimating content and composition of intracellular metabolites, namely triglyceride accumulation in oleaginous filamentous fungi. As a continuation of that research, in the present study HTS FTIR was evaluated as a unified method for simultaneous quantification of intra- and extracellular metabolites and substrate consumption. As a proof of concept, a high-throughput microcultivation of oleaginous filamentous fungi was conducted in order to monitor production of citric acid (extracellular metabolite) and triglyceride lipids (intracellular metabolites), as well as consumption of glucose in the cultivation medium.

**Results:**

HTS FTIR analyses of supernatant samples was compared with an attenuated total reflection (ATR) FTIR, which is an established method for bioprocess monitoring. Glucose and citric acid content of growth media was quantified by high performance liquid chromatography (HPLC). Partial least square regression (PLSR) between HPLC glucose and citric acid data and the corresponding FTIR spectral data was used to set up calibration models. PLSR results for HTS measurements were very similar to the results obtained with ATR methodology, with high coefficients of determination (0.91–0.98) and low error values (4.9–8.6%) for both glucose and citric acid estimates.

**Conclusions:**

The study has demonstrated that intra- and extracellular metabolites, as well as nutrients in the cultivation medium, can be monitored by a unified approach by HTS FTIR. The proof-of-concept study has validated that HTS FTIR, in combination with Duetz microtiter plate system and chemometrics, can be used for high throughput screening of microbial bioprocesses. It can be anticipated that the approach, demonstrated here on single-cell oil production by filamentous fungi, can find general application in screening studies of microbial bioprocesses, such as production of single-cell proteins, biopolymers, polysaccharides, carboxylic acids, and other type of metabolites.

**Electronic supplementary material:**

The online version of this article (10.1186/s12934-017-0817-3) contains supplementary material, which is available to authorized users.

## Background

Screening of a high number of candidate strains, as well as testing of different substrates and growth conditions, is a precondition for development and optimization of an efficient microbial bioprocess. Micro-bioreactors, usually in the form of multi-well microtiter plates, enable high-throughput parallel cultivation of microorganisms with culture volumes ranging from milliliter to nanoliter [1–7]. Application of such systems saves valuable time and decrease costs in the development of bioprocesses.

However, high-throughput screening can only be achieved if a high-throughput cultivation is followed by high-throughput measurement of biomass, intra- and extracellular metabolites, and substrate. Analysis is often performed by time-consuming approaches, that can involve two or more traditional analytical techniques in order to evaluate different type of analytes, thus significantly reducing the speed of the screening itself [6]. For example, screening of oleaginous microorganisms requires measurements of accumulation of intracellular lipids, as well as changes in chemical composition of the growth media. Usually this is obtained by tedious lipid extraction methods followed by gas chromatography (GC), while substrate consumption and release of extracellular metabolites is usually monitored by high performance liquid chromatography (HPLC) and by biochemical assays [1, 7, 8]. Although chromatographies are powerful methods for the analysis of metabolites, different mobile-solid phase configurations are needed for different type of analytes based on their molecular weight, solubility, polarity, and other parameters, thus often the change of a configuration or instrumentation is needed.

Over the past decade, mid-infrared (MIR) Fourier transform infrared (FTIR) spectroscopy has emerged as a powerful tool for screening, studying, and monitoring of biological processes. FTIR spectroscopy is fast and non-destructive biophysical method that detects molecular bond vibrations. Unlike traditional analytical methods, FTIR spectroscopy is not restricted to one specific cell characteristic. Given that FTIR is based on the measurement of many different spectral cell characteristics, the resulting spectrum is a precise signature of the overall chemical composition of a sample. FTIR spectra are highly reproducible and informative, and can be used both for identification purposes and for quantitative and qualitative analysis of cell’s chemical constituents such as lipids, proteins, carbohydrates and biopolymers. For instance, FTIR has been used for screening of microorganisms based on their content of lipids [8–10], biopolymers [11], or general biomass composition [12, 13]. Since FTIR spectroscopy is able to perform multi-analyte analysis and provide a broad spectrum of information, it is thus considered as an alternative to traditional analytical methods in high-throughput screening. FTIR techniques, such as attenuated total reflection (ATR) cells and probes, have already been assessed for on-line monitoring of bioprocesses, including substrate consumption and extracellular metabolite formation [14–22]. A number of studies have demonstrated that infrared ATR sensors are ideal instruments for monitoring of various compounds, such as glucose [15–19], fructose [20], lactose [14], starch [16], acetate [15, 16, 20], lactate [17], lactic acid [14], ethanol [18–20], and ammonium [20]. Unfortunately, measurement of biomass in a bioreactor by ATR sensors is impractical without complex modifications of FTIR instrumentation [22, 23], and thus quantitative on-line measurements of biomass have not been conducted yet.

In addition to on-line process monitoring, FTIR also offers high-throughput analyses of microbial bioprocesses by high throughput screening (HTS) system. The analysis is usually achieved by depositing biomass samples on a multi-well IR-light-transparent microplate [7–10, 24–26]. Several studies have shown a high correlation of HTS FTIR spectroscopy with traditional analytical techniques, such as GC, HPLC, and biochemical assays, for different types of bioprocesses [7, 9, 27, 28]. However, the majority of studies have been focused on quantitative analysis of biomass, while high-throughput studies of substrates and extracellular metabolites had very limited scope [27, 28]. Thus, the application of FTIR spectroscopy in high-throughput screening of microorganisms has not been fully explored, despite the fact that it can perform high-throughput multi-analyte quantification of both intra- and extracellular metabolites and substrate consumption.

Compared with other commercial high throughput micro-bioreactors, Duetz microtiter plate system (Duetz-MTPS) is simple and cost-effective system that offers very high number of parallel cultivations [29, 30]. However, the system is usually limited only to preliminary strain screening due to lack of process information [6]. In our recent study, FTIR spectroscopy was combined with Duetz-MTPS for the screening of oleaginous filamentous fungi [7]. It has been shown that HTS FTIR spectroscopic analysis of lipids in cell biomass correlates very well with GC analysis, and can be used for the prediction of total lipid and several groups of fatty acids (saturated, monounsaturated, and polyunsaturated) in fungal cell biomass [7]. Analogous to Duetz MTPS, HTS FTIR features microplate design well suited for automation systems [31], thus it is far more suitable for high-throughput screening than probe- or cell-based ATR FTIR setting. In the present study we evaluate HTS FTIR spectroscopy as a unified method for simultaneous quantification of both intra- and extra-cellular metabolites, as well as substrate consumption in high-throughput screening of microbial bioprocesses. Oleaginous filamentous fungi, namely *Mucor circinelloides*, *Umbelopsis isabellina* and *Penicillium glabrum*, were used as a model organisms. As previously reported, lipid production for all the studied fungal strains was relatively high, reaching 28–34% of lipid content of the biomass [7]. In addition, *Penicillium* is a good producer of organic acids [32–36].

## Methods

### Fungal strains

Three oleaginous filamentous fungi were used in the study: *Mucor circinelloides* VI 04473 (Norwegian School of Veterinary Science; Oslo, Norway), *Umbelopsis isabellina* UBOCC-A-101350 (Université de Bretagne Occidentale Culture Collection; Plouzané, France) and *Penicillium glabrum* FRR 4190 (Commonwealth Scientific and Industrial Research Organisation; North Ryde, Australia).

### Cultivation of fungi in high-throughput Duetz-MTP screening system

Cultivation in liquid medium was performed in the Duetz-MTP screening system (Enzyscreen, Netherlands), consisting of 24-square polypropylene deep well plates, low-evaporation sandwich covers, and extra high cover clamps. Duetz plates were mounted in two Innova 40R refrigerated desktop shakers (Eppendorf, Germany). The broth medium was prepared according to the protocol described in Kavadia et al. [37] with modifications (g L^−1^): glucose 80, yeast extract (total nitrogen 10.0–12.5%) 3, KH_2_PO_4_ 7, Na_2_HPO_4_ 2, MgSO_4_·7H_2_O 1.5, CaCl_2_·2H_2_O 0.1, FeCl_3_·6H_2_O 0.008, ZnSO_4_·7H_2_O 0.001, CoSO_4_·7H_2_O 0.0001, CuSO_4_·5H_2_O 0.0001, MnSO_4_·5H_2_O 0.0001. All chemicals were analytical grade (≥ 99%), and supplied by Merck (Germany), except yeast extract (Oxoid, England). Details of preparation of spore suspension and medium can be found in Kosa et al. [7]. In each well 2.5 mL of broth medium was inoculated with 50 µL of fungal spore suspension. Cultivations were performed for 12 days at 20 and 30 °C, at 300 rpm agitation speed (circular orbit 0.75” or 19 mm). Each day, one plate was removed from both shakers for analysis.

### Experimental design

All microplates were prepared in the following way: the first eight wells were inoculated with *M. circinelloides*, the second eight wells with *U. isabellina*, and the last eight wells with *P. glabrum*. For each strain, growth medium, supernatant of fermentation broth and biomass of the first three wells, considered as biological replicates, were used for FTIR and HPLC analyses. The merged biomass from the other five wells was used for lipid analysis by gas chromatography (GC), as described in Kosa et al. [7]. In total, 216 supernatant and 6 growth media samples were measured by FTIR and HPLC. Moreover, 210 and 70 biomass samples were measured by FTIR and GC respectively; biomass of *U. isabellina* and *P. glabrum* cultivated at 20 °C were not sampled on the first day due to insufficient growth.

### FTIR spectroscopy analysis

FTIR analyses of supernatant samples were conducted by both ATR and HTS accessories, while fungal biomass was analyzed by HTS only. Each sample was measured in three technical replicates, resulting in 648 spectra of supernatant and 18 spectra of growth media for both ATR and HTS, as well as 630 HTS spectra of biomass.

ATR measurement were performed using a Vertex 70 FTIR spectrometer (Bruker Optik, Germany) with the single-reflection attenuated total reflectance (SR-ATR) accessory. The ATR IR spectra were recorded with 32 scans using the horizontal SR-ATR diamond prism with 45° angle of incidence on a Specac (Slough, United Kingdom) High Temperature Golden Gate ATR Mk II. From each suspension or supernatant, 10 µL were transferred on the surface of the ATR crystal, and measured in three technical replicates. Spectra were recorded in the region between 7000 and 600 cm^−1^ with a spectral resolution of 4 cm^−1^. Each spectrum was recorded as the ratio of the sample spectrum to the spectrum of the empty ATR plate.

HTS measurements were performed from ten times diluted supernatant samples by using the High Throughput Screening eXTension (HTS-XT) unit coupled to the Vertex 70 FTIR spectrometer (both Bruker Optik, Germany) in transmission mode. The washed fungal biomass was sonicated, in order to prepare homogeneous suspension; the detailed procedure for preparation of the biomass has been described previously [7]. From each suspension or supernatant, 8 µL were transferred to an IR-light-transparent silicon 384-well microplate (Bruker Optik, Germany) in three technical replicates. Samples were dried at room temperature for 2 h to form films that were suitable for FTIR analysis. The spectra were recorded in the region between 4000 and 500 cm^−1^ with a spectral resolution of 6 cm^−1^ and an aperture of 5.0 mm. For each spectrum, 64 scans were averaged. Each spectrum was recorded as the ratio of the sample spectrum to the spectrum of the empty microplate.

### HPLC and pH analyses

Glucose and citric acid content of the starting growth media, as well as of the supernatant of fermentation broths, was quantified by an UltiMate 3000 UHPLC system (Thermo Scientific, USA), equipped with RFQ-Fast Acid H + 8% (100 × 7.8 mm) column (Phenomenex, USA), and coupled to a refractive index (RI) detector. Samples were diluted ten times before analysis, filter sterilized, and subsequently eluted isocratically at 1.0 mL min^−1^ flow rate in 6 min with 5 mM H_2_SO_4_ mobile phase at 85 °C column temperature. The pH measurements of growth media were conducted by a PHM210 MeterLab electrode (Radiometer Analytical SAS, France).

### Data analysis

For the analyses of ATR and HTS spectral sets of supernatant (including starting growth medium), spectral region of 1900–700 cm^−1^ was selected as this spectral region contains bands distinctive for both glucose and citric acid. ATR spectra were baseline offset corrected, and the obtained data set was used in the data analyses. HTS spectra were smoothed and transformed to second derivative form by Savitzky–Golay algorithm using a polynomial of power 2 with window size 15. Furthermore, the data set was reduced by taking an average of technical replicates, resulting in the data set with 222 spectra that was used in the data analyses. The description of the pre-processing of HTS spectra of biomass can be found in Kosa et al. [7].

Chemical similarities between samples were estimated by using principal component analysis (PCA), while partial least square regression (PLSR) was used to establish calibration models for glucose and citric acid. PLSR models were established by using a data set of HPLC reference measurements (responses) as a Y matrix, which was regressed onto an X matrix containing FTIR measurements (predictors). Optimal number of PLSR components (i.e. PLSR factors) of the calibration models (*A*_*Opt*_), root-mean-square error (RMSE) and coefficient of determination (R^2^) were calculated, and the optimal model was selected based on the lowest *A*_*Opt*_ having insignificantly higher RMSE than the model with the minimum RMSE. PLSR models for glucose prediction were based on measurements of all three fungal species, while the models for determination of citric acid were based on measurements of only *P. glabrum*. Model validation was performed using: (1) cross-validation (CV), where cross validation segments were defined per each strain, growth temperature, and day (74 segments in total, comprising either 9 spectra for ATR data set or 3 spectra for HTS set), and (2) independent test set validation (ITV), where PLSR models were built by excluding data on *P. glabrum* cultivated at 30 °C (540 and 180 spectra for determination of glucose by ATR and HTS respectively; 126 and 42 spectra for determination of citric acid by ATR and HTS respectively), and the data of *P. glabrum* 30 °C was subsequently used as an independent test set (108 and 36 spectra for ATR and HTS respectively). All pre-processing methods and data analyses were performed using The Unscrambler × 10.5 (CAMO Software, Oslo, Norway).

## Results and discussion

### Reference measurements of intra- and extracellular metabolites

**Fig. 1:**
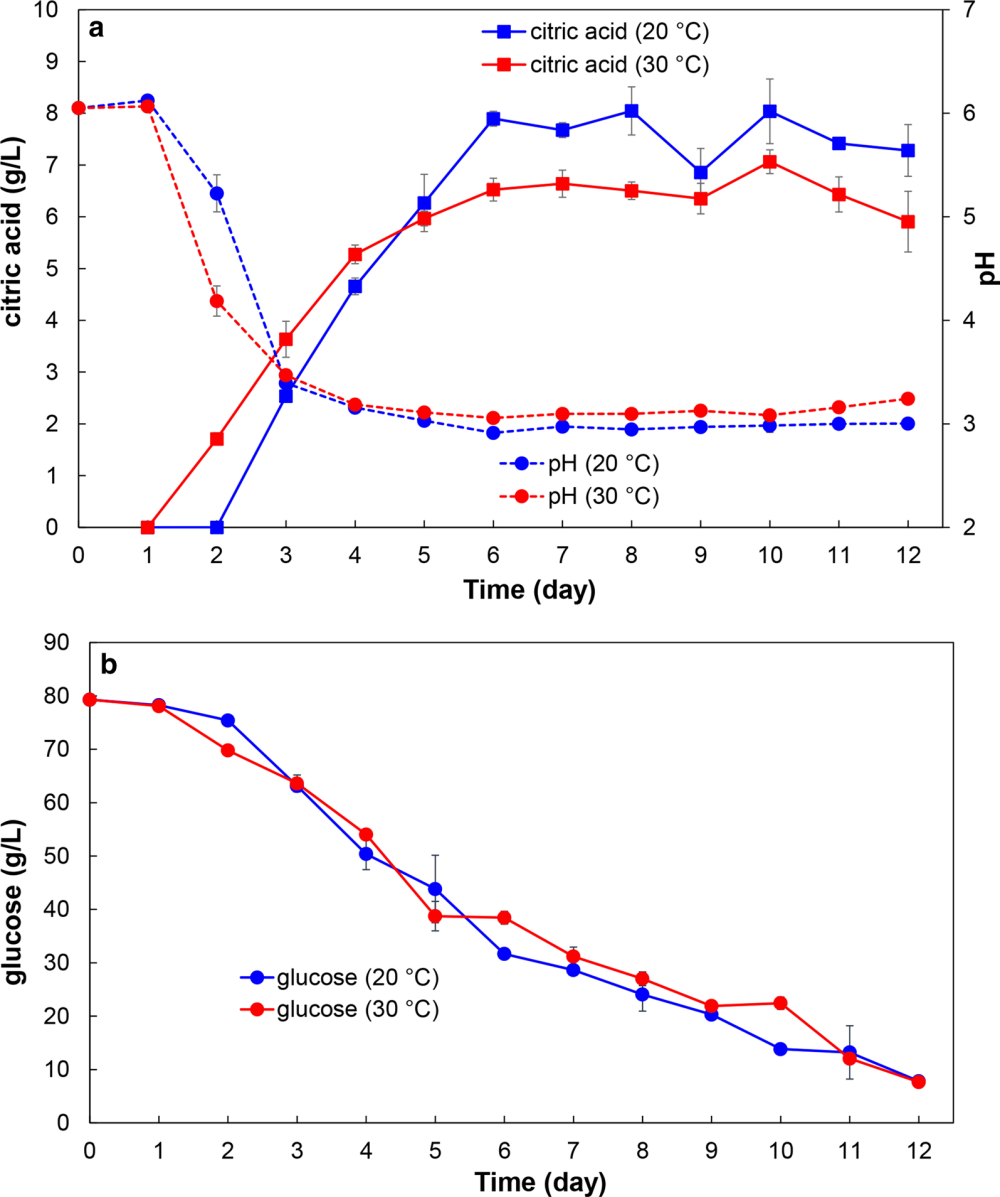
**a** Progression of pH values and citric acid concentration (obtained by HPLC measurements) in cultivation medium of *P. glabrum* at 20 and 30 °C. **b** Progression of glucose concentration (obtained by HPLC measurements) in cultivation medium of *P. glabrum* at 20 and 30 °C

Measurements of substrate (glucose) and extracellular metabolite (organic acids) in the growth media were performed by HPLC (Fig. 1), while lipid accumulation in the biomass was measured by GC as previously reported [7]. The pH measurements of growth media for *P. glabrum* cultivations have shown rapid drop of pH values, from pH 6 to 3 during the first 4 days of cultivation (Fig. 1a), while the pH values for growth media of *M. circinelloides* and *U. isabellina* have remained stable during the whole cultivation period. The drop of pH values was a result of simultaneous production of acids and lipids in *P. glabrum*. It has been reported previously that some oleaginous fungi can produce large amounts of organic acids, at the expenses of lipid accumulation, when grown under nitrogen-limited conditions [38].

HPLC measurements of organic acids and alcohols have shown significant citric acid production for *P. glabrum* cultivations, while productions of extracellular metabolites were negligible during the cultivation of *M. circinelloides* and *U. isabellina*. Citric acid production for *P. glabrum*, at 20 and 30 °C cultivation temperatures, has reached approx. 7.5 and 6.5 g L^−1^ respectively after 6 days of cultivation, and has remained stable for the subsequent 6 days (Fig. 1). In addition to citric acid production, the growth medium of *P. glabrum* has developed intensive yellow colour at the end of cultivation, however the chemical causing such colour change has not been identified.

### FTIR spectra of growth media and fungal biomass

**Fig. 2:**
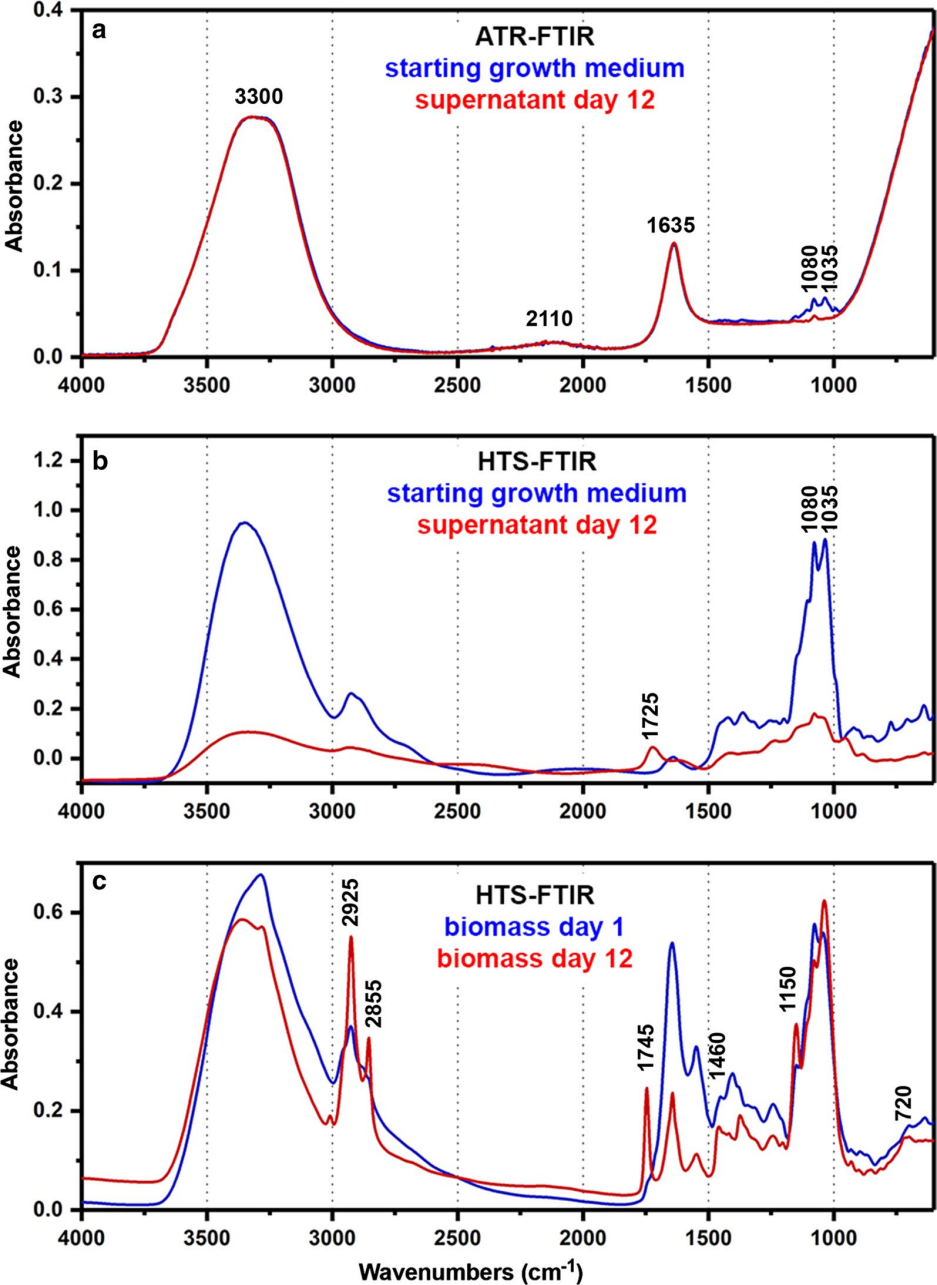
**a** ATR FTIR spectra of growth media, **b** HTS FTIR spectra of growth media, and **c** HTS FTIR spectra of biomass for *P. glabrum* cultivation at 30 °C. The marked bands are associated with molecular vibrations of (W) water, (G) glucose, (C) citric acid, and (L) lipids

The FTIR spectra of growth media show wide-ranging difference between the ATR and HTS spectra. The ATR spectra (Fig. 2a) are dominated by water vibrational bands at approx. 3300 (O–H stretching), 2110 (HOH bending + libration), 1635 (HOH bending), and 580 cm^−1^ (libration). The principal glucose bands at 1200–900 cm^−1^ (C–O–C stretch, C–OH stretch, COH deformation, COC deformation, pyranose ring vibrations) are noticeable in the ATR spectrum of growth media. These bands have much narrower profiles than the broad water bands, with full width at half-maximum for glucose vibrational bands being approx. 30–40 cm^−1^, compared to 100–400 cm^−1^ for water bands. The principal bands of citric acid, at 1725 (acid C=O stretch) and 1500–1000 cm^−1^ (C–O, C–OH, C–C vibrations) are mostly overlaid with stronger signals of either water or glucose in the ATR spectra. Since the samples for HTS FTIR measurements were recorded as dry films, the HTS spectra are largely devoid of water bands, and clearly show principal signals of both glucose and citric acid (Fig. 2b). The concentration of citric acid in the growth media during *P. glabrum* cultivation (0–0.046 M) was several orders of magnitude lower than glucose concentration (0.042–0.444 M), reaching parity only for the end-cultivation values. This is evident in the HTS spectra of growth media on the 12th day of cultivation (Fig. 2b), where citric acid peak at 1725 cm^−1^ is of the same magnitude as glucose peak at 1035 cm^−1^.

The development of cellular lipids can be easily detected in the biomass HTS FTIR spectra (Fig. 2c) by presence of peaks at 3050–2800 (C–H stretch), 1745 (ester C=O stretch), 1460 (CH_2_ bending), 1250–1070 (C–O–C stretch and deformation) and 720 cm^−1^ (CH_2_ rocking). The detailed FTIR spectral assignation of biomass for the all three fungal strains can be found in Kosa et al. [7].

**Fig. 3:**
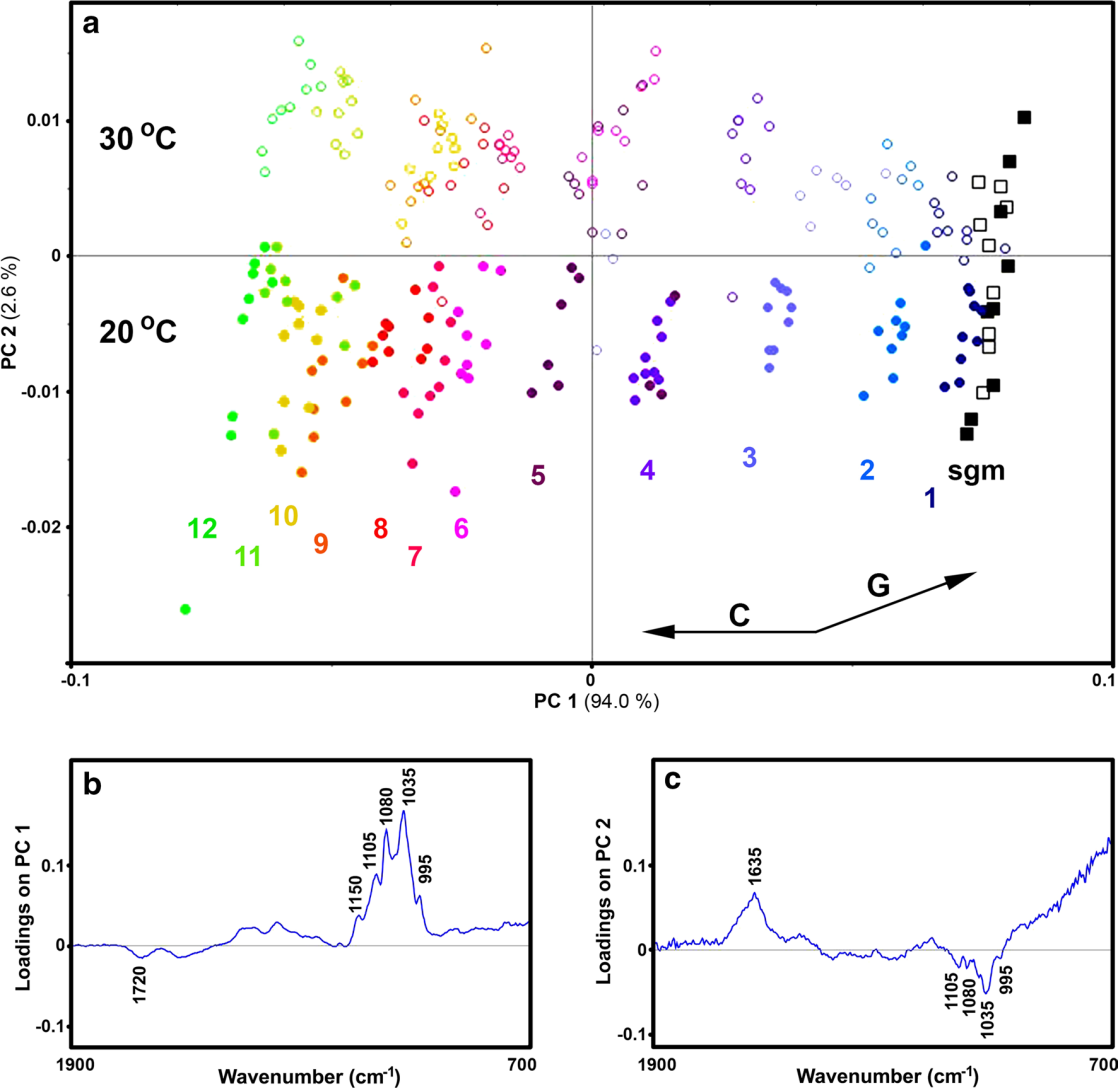
**a** PCA score plot of ATR FTIR spectral data set of cultivation medium of *P. glabrum* (baseline offset corrected spectra), with depiction of starting growth media (sgm), cultivation day (1–12), and temperature (dot/block—20 °C; circle/square—30 °C). C and G vectors approximate the increase in relative amount of citric acid and glucose respectively. The percent variances for the first five PCs are 94.01, 2.59, 0.98, 0.37 and 0.16. **b** and **c** Loading plots on the first two principal components of the PCA

**Fig. 4:**
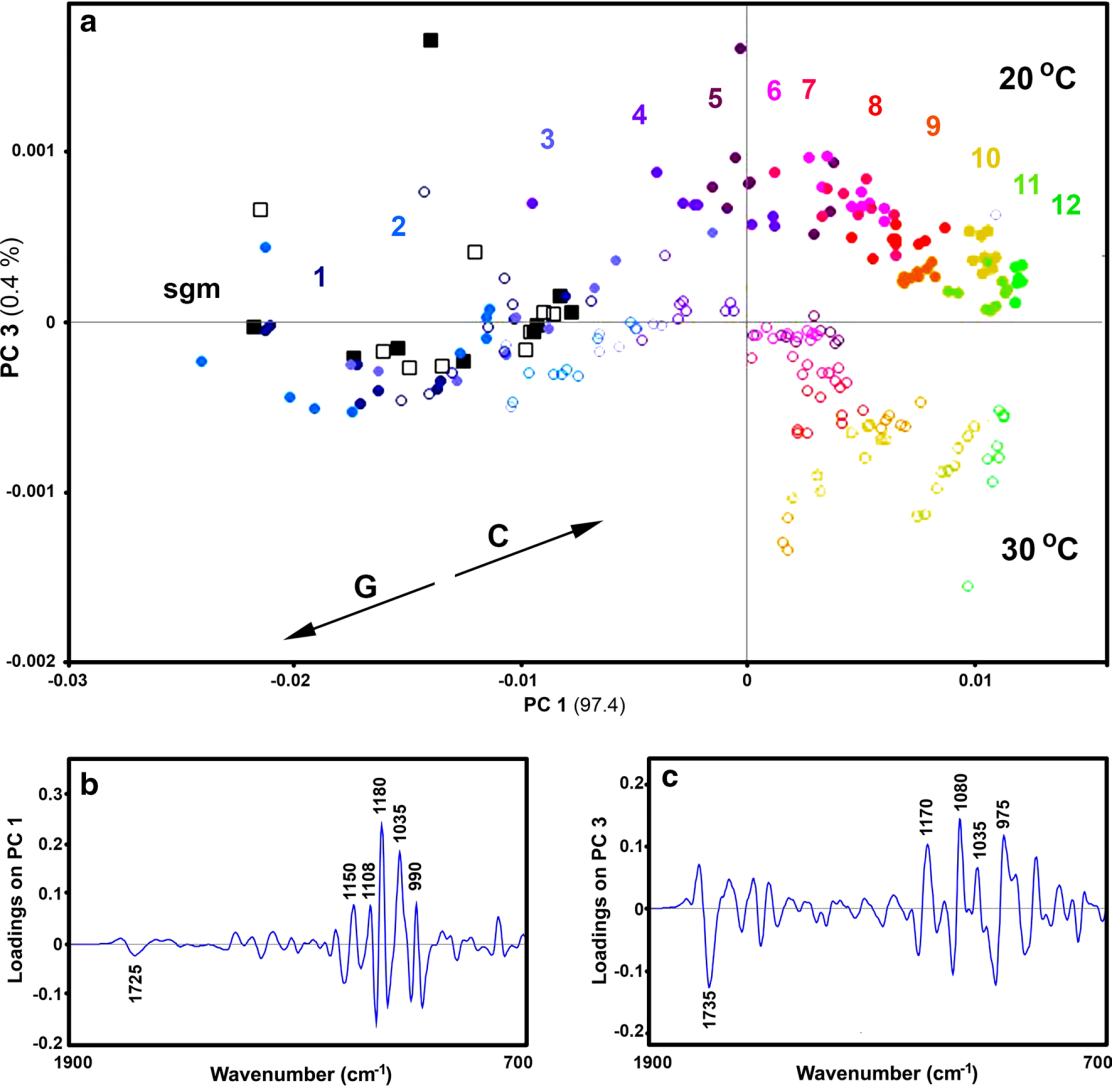
**a** PCA score plot of HTS FTIR spectral data set of cultivation medium of *P. glabrum* (second derivative spectra), with depiction of starting growth media (sgm), cultivation day (1–12), and temperature (dot/block—20 °C; circle/square—30 °C). C and G vectors approximate the increase in relative amount of citric acid and glucose respectively. The percent variances for the first five PCs are 97.45, 1.69, 0.36, 0.15 and 0.13. **b** and **c** Loading plots on the first and third principal components of the PCA

For both spectral data sets of growth media, the PCA results show that concentration change of glucose and citric acid during a cultivation can be monitored with great precision (Fig. 3). As can be seen in Figs. 3 and 4, for both ATR and HTS spectral data for *P. glabrum* cultivation, PC1 has high negative and high positive loadings related to glucose (1200–900 cm^−1^) and citric acid bands (1725 cm^−1^). Moreover, the influence of cultivation temperature on the chemical composition of growth media is evident even in the first couple of days of cultivation. In both data sets the score plots correctly indicate that the end-cultivation ratio of citric acid-to-glucose is higher at 20 °C than at 30 °C.

### Quantitative determination of glucose and citric acid in growth medium by FTIR spectroscopy

Quantitative estimates of glucose and citric acid in the cultivation media were obtained by PLSR analyses. The results show very high level of correlation between the FTIR and HPLC measurements for both HTS and ATR measurements (Tables 1, 2). The RMSE values for assessment of glucose by ATR for all three fungal species (RMSE = 5–6%) are consistent with the reported values for ATR cell and probe measurements of bacterial and yeast fermentations (RMSE = 6–12%) [16–19]. Likewise, the related glucose values for HTS measurement are consistent with the reported values for monitoring of mammalian cell cultures [27]. However, it should be noted that our study has covered one order of magnitude higher range of glucose concentration (up to 80 g L^−1^ glucose) compared to the one in the above mentioned study [27]. The only previous reported HTS measurement of glucose, where similarly high concentrations of glucose were measured in an artificial set of model solutions (and not an actual cultivation medium), has resulted with very large errors of prediction [28].

**Table 1:** PLSR coefficients of determination (R^2^) and root mean square errors (RMSE) for determination of glucose (for all three species), with the number of components in parenthesis (*A*_*opt*_); *CV* cross validation, *ITV* independent test validation

FTIR measurement mode	CV		ITV	
	R^2^ (*A*_*opt*_)	RMSE (g L^−1^ glucose)	R^2^ (*A*_*opt*_)	RMSE (g L^−1^ glucose)
ATR	0.98 (1)	3.59 (4.5%)	0.96 (2)	4.49 (5.6%)
HTS	0.96 (3)	4.87 (6.1%)	0.95 (2)	4.98 (6.2%)

**Table 2:** PLSR coefficients of determination (R^2^) and root mean square errors (RMSE) for determination of citric acid (for *P. glabrum*), with the number of components in parenthesis (*A*_*opt*_); *CV* cross validation, *ITV* independent test validation

FTIR measurement mode	CV		ITV	
	R^2^ (*A*_*opt*_)	RMSE (g L^−1^ citric acid)	R^2^ (*A*_*opt*_)	RMSE (g L^−1^ citric acid)
ATR	0.97 (5)	0.51 (5.8%)	0.88 (4)	0.76 (8.7%)
HTS	0.98 (5)	0.43 (4.9%)	0.91 (3)	0.75 (8.6%)

The number of components (PLS factors) used for building FTIR vs. HPLC calibration models for both FTIR techniques was low indicating high stability and reliability of the developed models. Moreover, the models for citric acid have low error even for independent test validation, where almost half of the data has been hold up. In addition to PLSR prediction models of the growth media, PLSR between gas chromatography fatty acid data and corresponding HTS–FTIR spectral data of biomass was used to set up models for the prediction of saturated, monounsaturated and polyunsaturated fatty acids, unsaturation index, total lipid content and principal fatty acids, and the results have been presented in Kosa et al. [7]. It has to be noted that the number of components used for building glucose and citric acid HTS–FTIR vs. HPLC calibrations was lower than FTIR–HTS vs. GC analysis of fungal lipids [7]. These results are logical since chemical complexity of the cultivation media (supernatant) is relatively low when compared with the fungal biomass.

### Comparison of FTIR–HTS and FTIR–ATR measurements

In general, the results for HTS measurements were very similar to the results obtained with ATR methodology, with comparable RMSE values for both glucose and citric acid estimates (Tables 1, 2). This demonstrates that chemical analysis of both cultivation media and biomass can be performed by HTS–FTIR approach. However, the main differences between ATR and HTS approaches are worth discussing in detail. First, HTS measurement of growth media often requires optimization of sample concentration, as was the case in this study where spectra were obtained from ten times diluted supernatant samples. This requirement is often absent in ATR measurements, as demonstrated by published studies where ATR probes and cells were directly placed in the fermenters [14–22]. However, measurement of diluted samples can be an advantage of HTS approach over ATR since smaller quantities of supernatant are needed for measurement. This could be of importance in microbioreactor screening studies, where culture volumes are often extremely limited.

Secondly, in ATR measurement of growth media there is a controlled optical path length, resulting in extremely reproducible spectral measurements of technical replicates. Thus, only minimal requirements for spectral pre-processing are needed. For example, in this study, only the baseline offset correction was applied on the ATR spectral data set. In contrast, HTS measurement of growth media is characterised by much larger variations between the spectra of technical replicates, due to the irreproducible film formation on the silicon microplates. Because of morphological differences (mainly area and thickness) between the dried films, the resulting HTS spectra can have relatively large variation in absorbance values. Usually, this is not a major problem since the spectra can be normalised by applying standard normal variate or extended multiplicative signal correction, as it was the case with the HTS spectra of biomass [7]. However, such internal normalization should be applied only if the spectral set is more or less invariant regarding the total absorbance. This was not the case for the growth media, where concentration of nutrients has decreased by two orders of magnitude, resulting in large change in total absorbance and spectral profiles between the starting and final cultivation spectra (Fig. 2b). For that reason, differences in optical path length in HTS measurements were minimised by averaging individual spectra of technical replicates, as well as by converting the spectra into derivative form. The PLSR results for estimating citric acid and glucose indicate that spectral pre-processing was sufficient. However, if this is not the case, one possible solution would be an addition of an internal standard. For instance, an internal standard with a simple spectrum comprising of just a few sharp bands, such as thiocyanate salts with sharp S–C≡N stretching band at approx. 2100 cm^−1^, could be utilized in peak normalization preprocessing [39]. In addition, we can expect that the use of a robotic system for sample preparation on silicone plates could increase the precision of HTS–FTIR measurement [31].

Thirdly, since dry films are used for HTS measurements, the water signal is weak and probably a more detailed fingerprint of biomolecules can be obtained compared with ATR approach. This could be of interest for detection and assessment of low-concentration chemicals, such as pigments. On the other hand, measurement of dry films may prevent detection of volatile compounds in the growth media, such as low-molecular-mass organic acids and alcohols.

Finally, compared to ATR, microplate design of HTS–FTIR setup is consistent with microbioreactor plate design, and thus it is well suited for high throughput screening. Therefore, while ATR setup is probably an optimal choice for industrial scale bioprocess control, HTS setup displays clear advantage in screening studies.

## Conclusions

One of the main challenge in using the high throughput micro-bioreactors has been the lack of information on cultivation parameters, including concentration profiles of reactants and products. Analyses of substrate and metabolites are often bottleneck activities, and thus can significantly hinder high throughput platforms. In our studies of lipid production of filamentous fungi, we have demonstrated that HTS FTIR, in combination with Duetz-MTPS, can be used for high throughput screening of microbial bioprocesses. Intra- and extracellular metabolites, as well as nutrients in the cultivation medium, have been monitored by a unified approach. When compared with ATR FTIR, which is an established method for bioprocess monitoring, HTS FTIR offers almost equivalent prediction of glucose and citric acid. In addition, both Duetz-MTPS and HTS FTIR have good potential for full automatization. In conclusion, it has been demonstrated that HTS FTIR spectroscopy can be used as a rapid and versatile analytical method for gaining insights on microbial bioprocesses. It can be anticipated that the approach, demonstrated here on single-cell oil production by filamentous fungi, can find general application in screening studies of microbial bioprocesses, such as production of single-cell proteins, biopolymers, polysaccharides, carboxylic acids, and other type of metabolites.

## Additional file

**Additional file 1.** FTIR and HPLC data.

## Abbreviations

ATR: attenuated total reflectance
Duetz-MTPS: Duetz microtiter plate system
HTS: high-throughput screening
FTIR: Fourier transform infrared spectroscopy
PLSR: partial least squares regression
